# Commentary: Rifabutin Resistance Associated with Double Mutations in *rpoB* Gene in *Mycobacterium tuberculosis* Isolates

**DOI:** 10.3389/fmicb.2017.02274

**Published:** 2017-11-27

**Authors:** Sandeep Sharma, Noton K. Dutta

**Affiliations:** ^1^Department of Microbiology, Perelman School of Medicine, University of Pennsylvania, Philadelphia, PA, United States; ^2^Department of Biochemistry and Molecular Biology, Thomas Jefferson University, Philadelphia, PA, United States; ^3^Department of Medicine, Center for Tuberculosis Research, Johns Hopkins University School of Medicine, Baltimore, MD, United States

**Keywords:** tuberculosis, rifabutin, RpoB, double mutations, rifampicin

Rifabutin (RFB), a member of rifamycin family, was approved by the FDA in 1992 and was recognized in 2009 as an essential medicine by WHO (World Health Organization, 2011; Crabol et al., 2016). Rifabutin is recommended for tuberculosis (TB) [including *Mycobacterium avium complex* (MAC)] treatment and has been used in patients coinfected with HIV/AIDS (Rukasha et al., 2016). Rifabutin exhibits fewer drug-drug interactions than rifampicin (RIF) and induces cytochrome_P450_ (CYP_3A4_) to a much lower degree than RIF (Zhang et al., 2011; Dutta and Karakousis, 2015). It is associated with lower MIC values than RIF for MAC, *Mycobacterium tuberculosis* (*Mtb*), *Mycobacterium leprae* and other non-tuberculous mycobacteria and it yields higher MIC values against actively growing Mycobacteria (Van Ingen et al., 2010; Aziz et al., 2017). Rifabutin is quite stable in stomach acid (pH 2–8) and antacids, and it is more lipophilic in nature than RIF and accordingly associated with much higher uptake in tissue and therefore distribution (Blaschke and Skinner, 1996).

Rifampicin and RFB both inhibit *Mtb* by acting on the β-subunit of the *rpoB* gene of DNA-dependent RNA polymerase (Dutta and Karakousis, 2017). It is important to characterize *rpoB* gene mutations and their connection with RIF and RFB resistance to better understand the phenotypic and molecular results noted in clinical settings. Rifampin Resistance-Determining Region (RRDR) in the *rpoB* gene in *Mtb* is significantly associated with mutations from codons 507–533 of *rpoB*, within an 81 bp fragment of *rpoB* gene. A high level of rifamycin resistance (RIF and RFB) is exhibited by strains with mutations in codons 526 and 531 [Minimum Inhibitory Concentration (MIC) of RIF >160 μg/ml; RFB > 5 μg/ml] (Jamieson et al., 2014). However, there are some other mechanisms that also contribute to RIF resistance to *Mtb*. Approximately 5% of *Mtb* RIF resistance isolates exhibited an unknown mechanism for resistance, suggesting that some alternative mechanism—such as the upregulation of the transmembrane protein (efflux pumps), reduced cell wall permeability, or inactivation of drugs—was at play (Louw et al., 2009).

The cross resistance to RIF and RFB is very common. Roughly 25% of RIF resistance isolates with a mutation in codon 516 remain susceptible phonetically to RFB at a critical concentration of 0.5 μg/ml (Uzun et al., 2002; Senol et al., 2005; Van Ingen et al., 2011). Rifabutin has been suggested to be rational alternative to treat MDR-TB and XDR TB, particularly associated with *rpoB* mutation (Sirgel et al., 2013).

Jing et al. (2017) investigated cross-resistance between RIF and RFB among clinical isolates of M*tb* from the National Tuberculosis Clinical Laboratory, Beijing Chest Hospital, China. The study considered 256 isolates and attempted to establish a relationship between MIC and *rpoB* gene mutations. The majority of isolates exhibited a single mutation in the *rpoB* gene, followed by a double mutation or no mutation in the *rpoB* gene in the clinical isolates. Therefore, a specific mutation in the *rpoB* gene was associated with RIF resistance and RFB susceptibility (Jamieson et al., 2014).

The data of Jing et al. (2017) also emphasize the use of RFB in the place of RIF because MIC for all of the resistant isolates exhibited lower RFB MIC compared with RIF. The data are consistent with previous findings (Jamieson et al., 2014; Berrada et al., 2016), which have demonstrated that the *rpoB* mutations S531L, H526D, H526R, H526C, and D516V confer phenotypical high-level resistance to both RIF and RFB; amino acid substitution at codons L511P, D516G/Y, S522L, H526Y/L/D/N, S531Q, and L533P was associated with phenotypic resistance to RIF and susceptibility to RFB (Rukasha et al., 2016). Roughly 5% of cases of *Mtb* resistance to RIF did not exhibit any mutation in the *rpoB* gene, suggesting some alternative mechanism for RIF resistance or a mutation in another part of the *rpoB* gene. Mutations in codon 511, 515, 522, and 533 of *rpoB* were associated with a low level of resistance, and the overexpression of certain transmembrane proteins (*Rv1258C* and *Rv2136c* in the presence of RIF) were also associated with RIF resistance (Siddiqi et al., 2004; Sharma et al., 2010).

There have been reports that *rpoB*-independent RIF resistance in *Mtb* is due to overexpression of transmembrane proteins (efflux proteins) and can be countered by using EPIs (De Vos et al., 2013) A large number of studies conducted by different groups have focused on the combination of existing drugs with some natural molecules to lower the MIC value *in vitro*. Since RFB has exhibited reduced toxicity and other pharmacological advantages over RIF in HIV/AIDS patients, it should be tested in combination with molecules reported to increase drug efficacy and possess potential immunomodulatory activity in a murine model.

The conventional drug susceptibility assay can provide appropriate results for detecting *rpoB* mutation, but the method is time consuming: it takes several weeks to generate a susceptibility profile. This prolonged duration can result in a delay in treatment and the generation of drug resistance over time. Furthermore, there have been reports about the compensatory mutation in *rpoA* and *rpoC* regions that improve the fitness cost in RIF-resistant *Mtb* strains (De Vos et al., 2013; Ali et al., 2015). Other advanced methods (sequencing the RRDR, GeneChip, Proteomics) and bioinformatics tools give new hope for the detection of drug-resistant TB in clinical settings, but all these tools are expensive and are out of reach of many TB management programs across the world.

The current study is interesting and will help in epidemiology studies about the burden of RIF and RFB resistance against *Mtb* in China and many other high TB burden countries. Furthermore, studies of this kind will be useful for developing combination treatment regimen effective against mycobacterial diseases based on *rpoB* gene mutations (Deshpande et al., 2017). Additional studies will yield a better understanding of mutations in *rpoB* gene and facilitate the analysis of drug resistance to RFB associated with double mutations in the *rpoB* gene.

## Author contributions

SS and ND have made substantial, direct and intellectual contribution to the work, critically reading an earlier version of this manuscript and approved it for publication.

### Conflict of interest statement

The authors declare that the research was conducted in the absence of any commercial or financial relationships that could be construed as a potential conflict of interest.
